# Does the egg capsule protect against chronic UV-B radiation? A study based on encapsulated and decapsulated embryos of cuttlefish *Sepia officinalis*

**DOI:** 10.1098/rsos.230602

**Published:** 2023-07-19

**Authors:** Luis MOLINA-CARRILLO, Yann Bassaglia, Gaëtan Schires, Laure BONNAUD-PONTICELLI

**Affiliations:** ^1^ UMR Biologie des Organismes et Ecosystèmes Aquatiques, Muséum National d'Histoire Naturelle, CNRS 8067, Sorbonne Université, Paris, France; ^2^ Université Paris Est Créteil-Val de Marne (UPEC), France; ^3^ Station Biologique de Roscoff, FR2424, CNRS-Sorbonne Université, Roscoff 29682, France

**Keywords:** cuttlefish, egg capsule, UVB radiation, development, gene expression

## Abstract

Although the egg capsule plays a crucial role in the embryonic development of cephalopods, its ability to protect embryos from Ultraviolet (UV) radiation is unknown. Our study evaluated the photoprotection mechanisms of *S. officinalis* to UV-B radiation and estimated the ability of the black capsule to act as a physical shield against it. Embryos with and without capsule and juveniles were exposed to four experimental UVB conditions for 55 days. The effects of different UVB doses were evaluated in terms of morphological abnormalities and differences in gene expression between each group. We observed that the development might be severely impaired in embryos exposed to UVB without capsule protection, and these effects were time- and UVB-dose-dependent. In addition, we found variations in gene expression levels (light-sensitive, stress response and DNA repair) in different tissues as a function of UVB doses. We suggest a relationship between morphological abnormalities and the limit of molecular regulation. These results suggest that the quantitative differences in expression are essential for defining the survivability of the embryo face to UVB. Thus, we demonstrated that the egg capsule could ensure successful embryonic development of the cuttlefish *S. officinalis* even at high doses of UVB.

## 1. Introduction

Ultraviolet (UV) radiation, especially UV-B radiation (280–320 nm), is one of the main stress factors in aquatic ecosystems attributed to climate change [1,2]. UVB radiation can have detrimental effects, particularly for species spawning in shallow water fully exposed to natural sunlight and UVB radiation [3]. Indeed, eggs are particularly vulnerable because of their inability to move actively, which may lead to UV-B exposure for extended periods. However, exposure to sunlight throughout evolution has led to strong selection pressure resulting in mechanisms of protection of the offspring from UVB radiation [4]. Several studies suggest that egg pigments, especially melanin, may act as a barrier by absorbing the harmful wavelength of light, especially UVB, thus protecting development [5,6]. In cephalopods, as in other oviparous species, the egg capsule may play a dual role in physical and chemical protection [7]. Our model, *Sepia officinalis,* is a semelparous cephalopod with an active nekton-benthic lifestyle and a direct development that resides mainly on sandy and muddy coastal bottoms (2–3 m depth) [8]. The female of *S. officinalis* surrounds the ovocyte before fertilization with numerous layers impregnated with melanin-containing ink: this constitutes the capsule surrounding the embryo (electronic supplementary material, figure S1). The eggs are attached to natural or artificial support in the intertidal area, where they are subject at low tide to large variations in temperature, low humidity and high amounts of sunshine (irradiance). The embryonic development of *S. officinalisis* within the egg involves 30 developmental stages grouped into five different phases: cleavage (stages 1–9), gastrulation (stages 10–13), organogenesis, flat stage (stages 14–18), organogenesis, extension stage (stages 19–22) and organogenesis, growth stage (stages 23–30) when the embryo has the general adult conformation [9]. The light sensitivity of *S. officinalis* has been demonstrated in the later stages [growth stage (stages 23–30)] of development [10,11]. Light can be life-saving and life-threatening depending on its wavelength, exposure time, and intensity. In the tissues/cells of many organisms, light perception and UVB perception is made possible by various light-sensitive molecules, such as opsins and cryptochromes, which confer to the animal the ability to become sensitive to a broader range of wavelengths through the enhancement of these molecules [12]. In a recent study conducted in our laboratory, photosensitizing molecules such as arrestins, cryptochromes and opsins were identified in *Sepia officinalis* embryos in different organs [ocular such as eyes, and extra-ocular such as skin, and central nervous system (CNS)], suggesting the implementation of a light-sensitive system at early developmental stages [10].

Some animals can detect visible light but also UVB light. This ability of cephalopods has not been fully explored. However, it is well known that the effects of UVB radiations at molecular levels trigger many signalling pathways and repair mechanisms at the cellular level [13]. UVB rays have higher energy than visible wavelengths of light [14], and an excessive amount of UVB can induce free radicals, especially reactive oxygen species (ROS) [15]. The production of ROS is the main mechanism by which DNA damage can occur. The synergistic effects between UVB and ROS can cause extensive DNA damage and lead to apoptosis or cell death [16]. Enzymatic activities of superoxide dismutase (SOD), glutathione-S-transferase (GST), glutathione reductase, glutathione peroxidase (GPx) and catalase (CAT) play an essential role in the detoxifying of ROS into less reactive products [17,18]. Oxidative stress may also activate heat shock proteins (HSPs), especially the HSP70, which play vital roles in protein quality control and in repairing denatured proteins and provide a protective mechanism after exposure to stress [19,20]. Herrera-Vásquez *et al*. [21] and Zhou *et al.* [22] suggest that HSPs and GSTs support ROS processing systems initiated by antioxidant enzymes such as SOD, playing an essential role in the control of ROS levels and oxidative damage in the tolerance response to UVB light and photooxidative stress.

UV-B radiation can also indirectly alter DNA [13]. The resulting photolesions may occur whatever the level of DNA compaction, but their frequency depends on DNA sequence and the presence of DNA-associated protein, such as nucleosomal histones (i.e. H3, H4, H2A and H2B) or transcriptions factors [23,24]. These photolesions trigger DNA repair mechanisms, especially the main DNA repair pathway, nucleotide excision repair (NER). In addition, the transcription factor p53, essentially known for its role in cell cycle regulation, is also a key player in this cellular response [13,25,26].

In the present study, we estimated the role of the black capsule as physical protection against UV-B radiation and the molecular mechanisms involved in the photoprotection of *S. officinalis* embryos. We experimentally exposed eggs of *S. officinalis*, with or without their black capsule, to four different UVB doses and their effects were evaluated by: (i) recording mortality rate and morphological abnormalities and (ii) determining the transcriptional regulation of light-sensing (Sof_r-Opsin1, Sof_Cry6, Sof_Cry123), stress response (Sof_Sod3, Sof_Gst1, Sof_Hsp702) and DNA repair proteins (Sof_H2b5, Sof_p53) by digital PCR (dPCR). The results of this study provide new insights into the adaptation of *S. officinalis* to UV-B stress towards physiological tolerance and molecular control, as well as the role of the black capsule in limiting UV-B effects.

## 2. Material and methods

### 2.1. Biological samples and experimental design

Clusters of *S. officinalis* eggs came from the channel sea coast in Roscoff, France. One thousand and two hundred eggs around the same stage with their natural black egg envelope were collected and kept for two weeks in an open circulatory system with filtered sea water at 17°C under natural photoperiod conditions.

The embryonic development of *S. officinalis* is described with 30 stages [9]. The beginning of experimental UVB exposure started at stages 24–25, just before the beginning of the eye pigmentation [9] when light-sensitive structures come into play [10] (electronic supplementary material, figure S1).

At stage 24, the black capsule was removed with forceps for the concerned eggs (600), leaving the transparent chorion and the perivitelline fluid surrounding the embryo still in place. Eggs with black capsule (BC) and without capsule (WC) were randomly and equitably distributed into 2 × 12 20 l-glass tanks (electronic supplementary material, figure S2). Each tank (in quadruplicate) was subjected to one of the four UVB radiation conditions. The UVB exposure trial lasted for 55 days.

The following conditions were kept throughout the whole experiment: water temperature around 17°C (17.3 ± 0.6°C), salinity around 35°C (35.6 ± 1.4) and pH around 8 (8.1 ± 0.06). In addition, ammonia, nitrite and nitrate levels were checked weekly through colourimetric tests (Macherey-Nagel) and kept below the detection limits.

### 2.2. Exposure experiments and sampling

The UVB irradiances and daily doses applied in this study were based on the data collected *in situ* (egg-laying area; 48° 43′ 40.1″ N, 03°58'28.2″ W, Coast of Roscoff, France) and the data available in different coastal areas [27] (electronic supplementary material, table S1). *Sepia officinalis* eggs were exposed to the following experimental radiation conditions: PAR (400–700 nm, No-UVB) and PAR + UVA + UVB (280–700 nm). The former was used as the control, while the latter was subdivided into three different UVB doses: (1) low UVB (PAR + UVA + UVBL = UVB-L, 18.6 µW cm^−2^), (2) moderate UVB (PAR + UVA + UVBM = UVB-M, 33.4 µW cm^−2^), and (3) high UVB [PAR + UVA + UVBH = UVB-H, 57.6 µW cm^−2^)] (electronic supplementary material, table S2). The spectral irradiance was determined with a Spectrometer STS-UV (Ocean Insight Co, Orlando, FL, USA). A lighting system using T5 (2, 6 and 12%UVB/30%UVA) 39W/88CM UVB (RP L.T.D., Wakefield, UK) was designed to simulate the daily UVB doses, and the lamps T5 0.0UV-STOP 9W849MM were used as control (electronic supplementary material, figure S3). The eggs were exposed daily for 12 h to the four different UVB conditions. The effect of different cumulative radiation dosages was tested by sampling embryos (BC and WC) for morphological analysis on days 5 (stage 24/25), 12 (stage 27/28), 24 (stage 30 early) and 36 (stage 30 late) before hatching. After hatching, the juveniles were free to move; they left the basket and were positioned preferentially at the bottom of the tank. They were sampled on days 45 [9 days after hatching (dah)], 49 (13 dah) and 55 (19 dah). For each sampling point, eight embryos/juveniles were randomly collected from each experimental tank (*n* = 32 per treatment). Embryos were extracted from the chorion in filtered seawater on ice to anaesthetize the animals; each embryo/juvenile was staged [9] and observed to evaluate the presence of morphological abnormalities using a standard stereomicroscope (Zeiss, Germany, Software ZEN 3.3). Mortality was recorded throughout the experiment.

The morphological abnormalities and lesions were described by comparison with the control group (NUVB) according to the following criteria: (a) localization of morphological abnormalities and lesions (anatomical regions), (b) severity of morphological abnormalities and lesions. In addition, the percentage of embryos/juveniles showing morphological abnormalities and lesions was determined.

For gene expression studies, the samples were immediately immersed in RNA later and kept in RNA later (SIGMA) at −80°C before being studied.

### 2.3. Extraction, DNase treatment and reverse transcription

Three embryos/juveniles on days 12 (stage 27/28), 24 (stage 30) and 49 days (juveniles 13 dah) were used per biological condition. The eyes were dissected, the lens was removed, and the brain, optic lobes and dorsal skin were dissected. Total RNA was extracted using the NucleoSpin R.N.A. midi kit (Macherey Nagel) following the manufacturer's protocol, treated according to the Ambion Turbo DNA-free Kit (Ambion, Applied Biosystems, Darmstadt, Germany), and cleaned with NucleoSpin R.N.A. Clean-up (Macherey Nagel). Quantity and quality were assessed with Qubit 3 fluorimeter (Invitrogen) and BioAnalyzer 2100 (Agilent). RNA integrity was confirmed by a 1.2% denaturant agarose-formaldehyde gel. RNA was diluted to a final concentration of 400 ng µl^−1^and stored at −80°C before use.

Single-strand cDNA was synthesized with SuperScript III First-Strand Synthesis System kit for RT-PCR (Invitrogen, Carlsbad, CA, USA), following the manufacturer's instructions.

### 2.4. Primers design and selection

We designed eight primers sets (electronic supplementary material, table S3) from EST's database (www.ncbi.nlm.nih.gov/dbEST/) and sequences previously reported for *Sepia officinalis* and *Sepia maindroni* from the NCBI GenBank database. The primers were designed using Primer 3 software [28] and tested using cDNA from cuttlefish eyes, skin and CNS (CNS = brain and optic lobes). The PCR mix includes REDTaq, PCR Reaction Mix (Eurogentec, Seraing, Belgium) and 10 µM of each primer in a final volume of 50 µl. The thermocycler program was: 5 min at 95°C, 40 cycles of 30 s to 95°C, 1 min to 58°C, 30 s at 72°C and the final extension of 2 min at 72°C. For the visualization of PCR products, 2% agarose gel was used.

### 2.5. Gene expression analysis using digital PCR

A QIAcuity Digital PCR System (Qiagen, Hilden, Germany) was used to perform absolute quantification of gene expression by using the QIAcuity EG PCR Kit (Cat No. 250113; Qiagen) and 8.5 K 96-well Nanoplates (Cat No. 250021; Qiagen). The QIAcuity 8.5 K 96-well Nanoplates are microfluidic dPCR plates that process 96 samples with up to 8.5 K partitions/well. The PCR reaction occurred in each partition, and the partition volume was 0.34 nl. The dPCR analyses were performed in a final volume of 12 µl comprising 4 µl of 3 × EG PCR Master mix buffer, 1 µl of primers (5 µM forward primer, 5 µM reverse primer), 5 µl of RNase-free water and 2 µl of template cDNA. The conditions for dPCR were as follows: 1 cycle at 96°C for 2 min, followed by 40 cycles of 15 s at 95°C, 30 s at 58°C and 15 s at 72°C, with a final cooling step for 5 min at 40°C. Three dPCR replicates were analysed for each sample. The initial copy value was automatically calculated by the QIAcuity Suite Software V1.1.3 193 (Qiagen, Germany), and quantities were exported as Copies/μl of the reaction. The dPCR assays were performed using automatic settings for threshold and baseline. dMIQE checklists are provided in electronic supplementary material, tables S4–S6 and figures S4 and S5.

### 2.6. Statistical analysis

The results of mortality and malformation rates were presented as mean ± s.e.m. Gene expression data were analysed and visualized using Statsmodels (Python module) O.L.S. [29] to detect significant differences between the treatments (UVB-L and UVB-M) and between groups (BC and WC). Statistical difference was determined using Student's *t*-test, and the significance level was set at *p* < 0.05.

## 3. Results

### 3.1. UVB treatments

The moderate and high irradiances of UVB applied in the present experiment were 33.4 ± 0.04 for UVB-M and 57.6 ± 0.07 µW cm^−2^ for UVB-H, representing a daily dose of the embryos 14.4 kJ cm^−2^ and 24.8 kJ cm^−2^, respectively.

**Figure 1.**
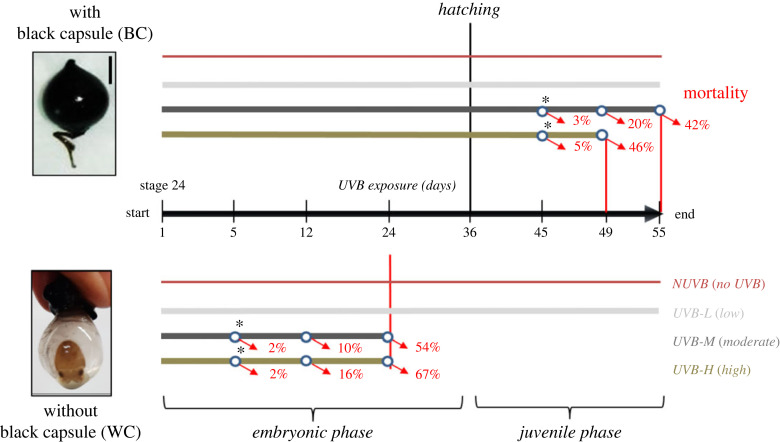
Mortality rate chronology of cuttlefish embryos/juveniles with capsule (BC) and without capsule (WC) exposed to different doses of UVB (UVB-L, UVB-M and UVB-H) and without UVB (NUVB). Mortality was recorded at 5, 12, 24, 45, 49 and 55 days of UVB exposure. * Asterisks represent the beginning of morphological abnormalities.

Only 1–5% mortality was observed in embryos with capsule (BC) and without capsule (WC) exposed to the NUVB and UVB-L treatments. However, embryos without capsule showed a higher and earlier mortality rate than embryos with capsule under UVB-M (54%) and UV-H (67%) treatments. Therefore, an endpoint was determined for the WC group at 24 days of exposure to the UVB-M and UVB-H treatments (figure 1).

In the BC group, mortality begins only in juveniles after 45 days of UVB exposure (9 days after hatching). Severe morphological abnormalities and skin lesions were observed with a high mortality rate with UVB-M (42%) and UV-H (46%). The endpoint was identified at 55 days of UVB exposure (19 days after hatching) to the UVB-M treatment and 49 days of UVB exposure (13 days after hatching) to the UVB-H treatment (figure 1).

### 3.2. Morphological phenotypes observed in embryos/juveniles exposed to UV-B radiation

#### 3.2.1. Embryos

**Figure 2.**
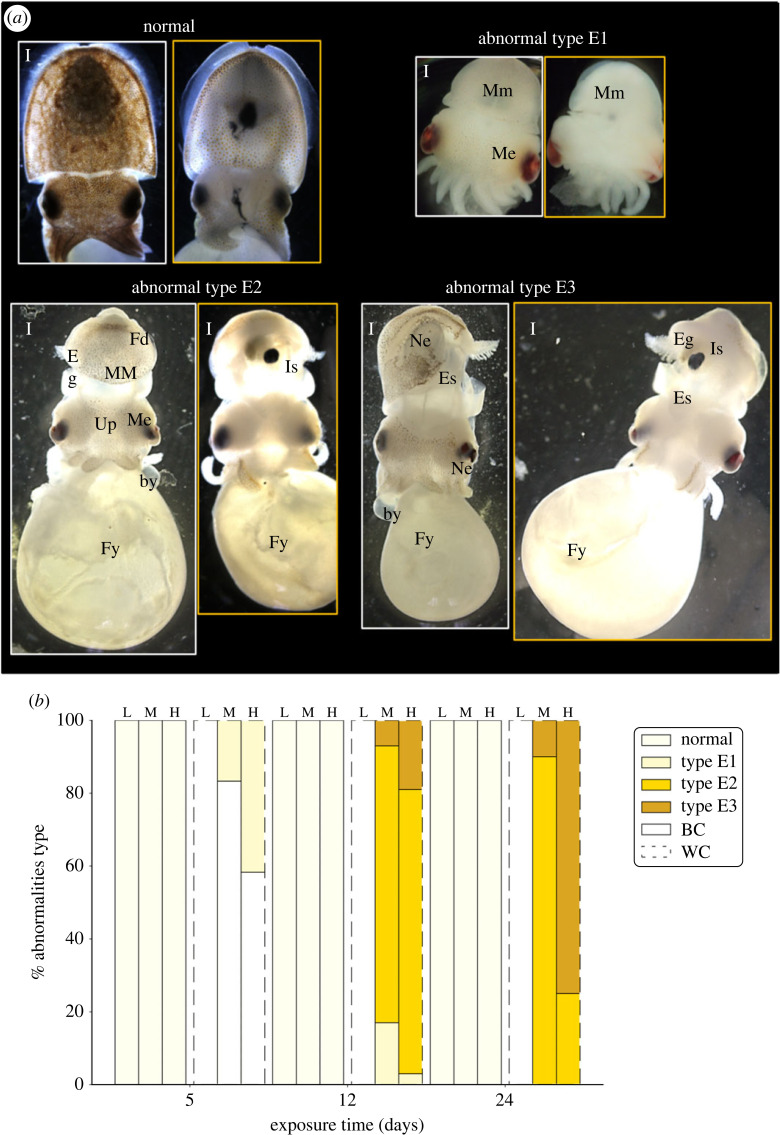
Morphological abnormalities observed in irradiated *S. officinalis* embryos during early development. (*a*) Embryos were categorized as normal [‘normal’ shape, healthy appearance], abnormal type E1 [reduced embryonic size, mild malformed mantle and eyes (Mm and Me)], abnormal type E2 [underdeveloped mantle (MM) and fin (Fd) exposing the gills (Eg) and the ink sac (Is), malformed eyes (ME) with untypical shape, hypopigmented skin with untypical pigment (Up) dispersion/opaque appearance, fissures (Fy) and blisters (by) in the yolk], abnormal type E3 [complete body deformity, epidermal sloughing (Es), necrosis (Ne) in dorsal mantle tissue and eyes, fissured yolk (Fy)]. Scale bar: 1 mm. Images in white boxes correspond to the dorsal position and in yellow boxes to the ventral position. (*b*) Percentage of each type of abnormalities observed in embryos with black capsule (BC) (solid line) and without capsule (WC) (dotted line), after 5, 12 and 24 days of UVB exposure [UVB-L (L), *n* = 32; UVB-M (M), *n* = 31; UVB-H (H), *n* = 32].

On visual examination, we did not observe any morphological differences between the NUVB and UVB-L treatments throughout the experiment; in the WC and BC groups, the embryos exhibited a regular shape with a healthy appearance. By contrast, embryos without capsule (WC) showed obvious abnormalities under UVB-M and UVB-H treatments compared with control embryos not exposed to UVB. These effects of UVB treatment on embryonic phenotype have been quantified by categorizing embryos as ‘normal’ or abnormal at the E1, E2 or E3 level using morphological criteria (figure 2*a*), which are consistent with those used by several authors in cephalopods and fish [27,30,31].

Increasing doses of UVB irradiation increased the percentage of embryos with morphological abnormalities and evidence of skin lesions (sunburn). This effect was detectable after 5 days of exposure in WC embryos (17% of abnormal type E1 with UVB-M treatment and 42% with UVB-H treatment) and reached 100% of morphological abnormalities on day 12 of exposure. Finally, after 24 days of exposure, cumulative adverse effects were observed in WC embryos after UVB-M and UVB-H treatments with 90% and 25% of abnormal type E2 and 10% and 75% of abnormal type E3, respectively. Significantly, BC cuttlefish embryos showed a normal shape with a healthy appearance throughout the embryonic phase regardless of the different UVB doses (UVB-L, -M and -H) (figure 2*b*).

#### 3.2.2. Juveniles

**Figure 3.**
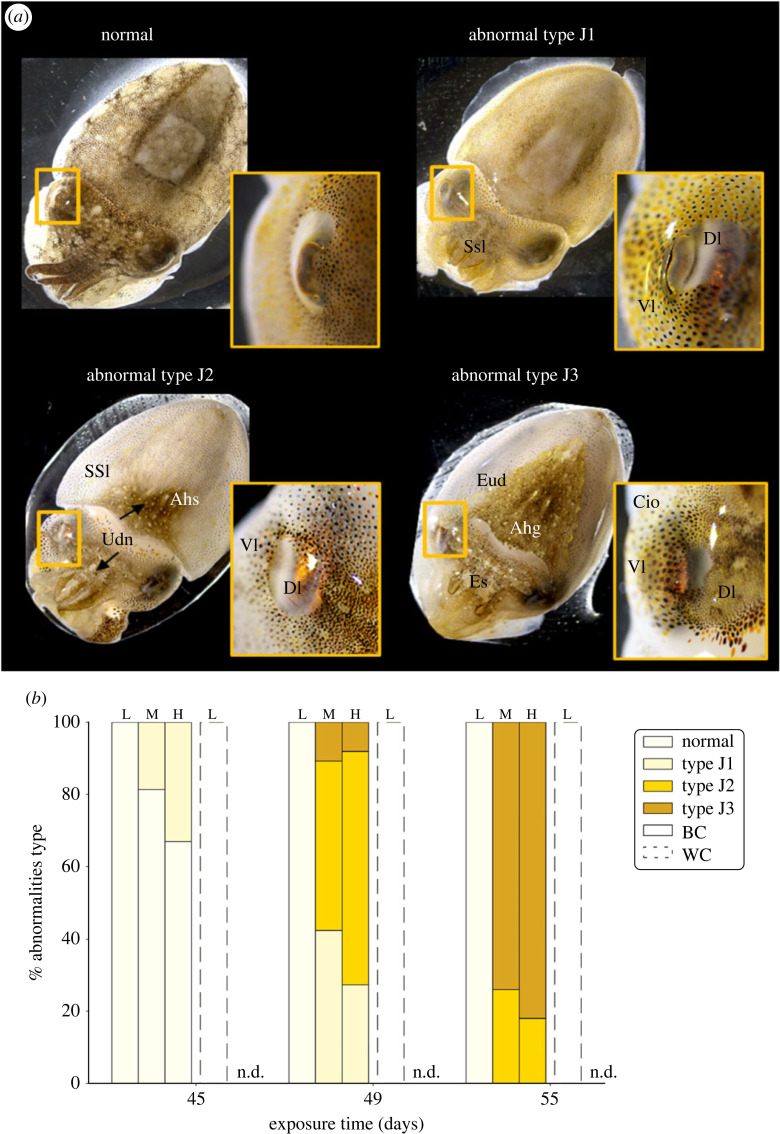
Morphological abnormalities observed in irradiated S. officinalis juveniles. (*a*) Juveniles were categorized as normal [no external abnormalities], abnormal type J1 [slight skin lesions (Ssl), located mainly on the head; complete retraction of the dorsal lid (Dl); slight malformed ventral lid fold (Vl)], abnormal type J2 [severe skin lesions (SSl) or ulcerative dermal necrosis (Udn) located mainly on the head and dorsal mantle; areas of atypical pigmentation (Ahp) with localized hyperpigmented skin; severely malformed dorsal lid fold with partial invagination or atrophy of eyes], abnormal type J3 [highly severe ulcerative dermal necrosis (EUd) and epidermal sloughing (Es); complete ocular invagination (Cio); generalized atypical pigmentation (Ahg) with hyperpigmented]. (*b*) Percentage of each type of abnormalities observed in juveniles that were developed in the capsule (BC) (solid line) and without capsule (WC) (dotted line), after 45, 49 and 55 days of UVB exposure (UVB-L (L), *n* = 32; UVB-M (M), *n* = 32; UVB-H (H), *n* = 30).

Similarly, the effects of UVB treatments on the phenotype were quantified in juveniles (*n* = 32) from each treatment, using a categorization as ‘normal’ or abnormal at the J1, J2 or J3 level using morphological criteria (figure 3*a*).

During the juvenile phase, we did not observe any morphological differences between NUVB and UVB-L treatments after hatching. In WC and BC groups, juveniles exhibited a regular shape with a healthy appearance. The abnormalities were observed only under UVB-M and UVB-H treatments. No data could be collected in the WC group due to high mortality in the UVBM and UVBH treatments during the embryonic phase. In the BC group, which showed no abnormalities before hatching, morphological abnormalities began to appear 9 days after hatching (45 days of UVB exposure), with only abnormalities type J1 observed (19 and 33% in UVB-M and UVB-H treatment, respectively) (figure 3*b*). On the 13th day after hatching (49 days of UVB exposure), more severe abnormalities were observed: UVB-M showed abnormal type J1 (42%), abnormal type J2 (47%) and abnormal type J3 (11%). UVB-H showed abnormal type J1 (27%), abnormal type J2 (65%) and abnormal type J3 (8%). Finally, cumulative adverse effects were observed 19 days after hatching (55 days of UVB exposure). UVB-M showed abnormal type J2 (26%) and abnormal type J3 (74%), and UVB-H showed abnormal type J2 (18%) and abnormal type J3 (82%) (figure 3*b*). Increasing doses of UVB irradiation increased the percentage of juveniles with morphological abnormalities and the severity of these abnormalities.

### 3.3. Gene expression analysis

Embryos under UVB-H treatment were not considered for gene expression analysis because of the highly severe effect on the animals evidenced at the morphological level. These morphological changes (atrophy of eyes, dissymmetry of brain parts and optic lobes, and highly severe ulcerative dermal necrosis) prevented the dissection of organs needed for relevant comparison. By contrast, the molecular effects of UVB light could be quantified in four different tissues (brain, eyes, optic lobes and skin) in animals exposed to NUVB, UVB-L and UVB-M in the presence (BC) or the absence (WC) of the black capsule and during three exposure times (12, 24 and 49 days) by measuring the expression level of three categories of genes (light-sensitive, stress response and DNA repair).

#### 3.3.1. Light-sensing molecules

**Figure 4.**
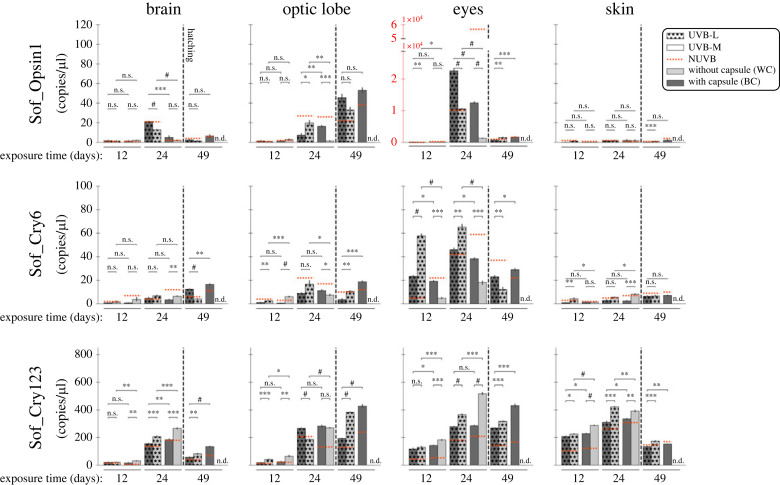
Gene expression levels (copies/µl, absolute quantification dPCR) of the light-sensing related genes (Sof_Cry123, Sof_Cry6 and Sof_r-Opsin1) in four different tissues (brain, eyes, optic lobes, and skin) from cuttlefish embryos/juveniles with black capsule (BC) and without capsule (WC), exposed to two doses of UVB light (UVB-L and UVB-M), at 12, 24 and 49 days after UVB irradiation. No-UVB irradiated animals were used to determine the relative baseline (NUVB), represented by the dotted red line. Statistical significance was determined by Student's *t*-test (*n* = 3; *p < 0.05, **p < 0.01, ***p < 0.001, ^#^*p* < 0.0001, n.s. = no significative, n.d. = no data). Note that the expression level of the Sof_r-Opsin1 gene in the eyes is two orders of magnitude higher than in the other tissues, represented by red colour.

The response of light-sensing molecules to UVB light exposure was evaluated with opsin (Sof_r-Opsin1) and cryptochromes (Sof_Cry6 and Sof_Cry123) (figure 4).

The expression level of Sof_r-Opsin1 was the lowest among the light-sensing genes studied here in all organs except in the eyes, where the expression level was a thousand times higher than in the brain or optic lobes. The reference level (NUVB = no UV) increased during eye development, and this was markedly amplified in the absence of capsule (WC, 24 days of exposure: 57 186.1 ± 4857 copies µl^−1^). In eyes, a significant difference was observed between UVB-L and UVB-M treatments, but in each condition, the absence of capsule decreased Sof_r-Opsin1 expression. Thus, the expression level of Sof_r-Opsin1 in eyes decreased with the amount of UVB (NUVB > UVB-L > UVB-M) during the embryonic phase. After hatching, the expression was low but remained higher than in other organs (1075 ± 168 copies µl^−1^). In the other organs, the expression level was very low, especially in the skin, whatever the experimental conditions. Surprisingly, by comparison with the other organs, the expression level of Sof_r-Opsin1 in optic lobes was two-fold higher in juveniles than in embryos.

As for Sof_r-Opsin1 expression, Sof_Cry6 appeared to be less expressed in the skin; even if significant differences were evidenced in the embryo between the treatments, no trend could be identified. By contrast, Sof_Cry6 was more highly expressed in the eyes than in the other organs. In the eye, the reference expressions (NUVB) were constantly higher in decapsulated embryos; nevertheless, they increased during development in decapsulated as well as in capsulated embryos. As for Sof_r-Opsin1, the expression of Sof_cry6 was higher under UVB-L conditions than in the absence of UV, especially at 12 days. Despite the low expression level, a significant inhibitory effect of UVB was constantly evidenced in embryos, especially in the absence of the capsule. In juveniles' eyes, the expression of Sof_Cry6 decreased and, surprisingly, was higher in hatchlings from capsulated embryos treated with UVB-M. In other organs, the expression was very low.

Unlike the previous genes, Sof_Cry123 was expressed at comparable levels in all four organs; the lowest levels were in the brain. In all organs, the reference expressions (NUVB) increased during development and decreased after hatchling in all conditions but in optic lobes from juveniles issued of decapsulated embryos under UVB-L. In all cases, the expression level under UVB exposure is higher than the reference expression; in UVB-exposed embryos, it was higher under UVB-M than UVB-L. Thus, the expression level of Sof_Cry123 increased with the amount of UVB (NUVB < UVB-L < UVB-M) during the embryonic phase. This UVB-induced up-regulation was particularly visible in eyes and optic lobes (24 and 48 days); the only exception was the skin of decapsulated embryos after 24 days of UVB.

#### 3.3.2. Stress response genes

**Figure 5.**
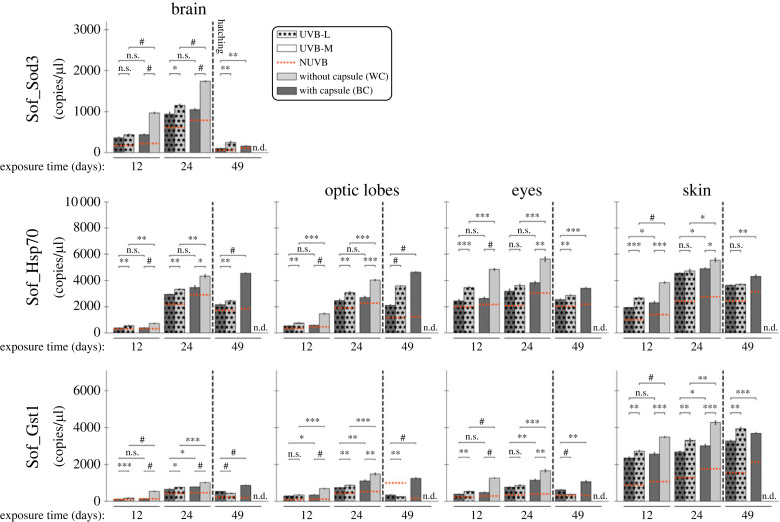
Gene expression levels (copies/µl, absolute quantification dPCR) of the stress response genes (Sof_Sod3, Sof_Hsp70 and Sof_Gst1) in the four different tissues (brain, eyes, optic lobes and skin) from cuttlefish embryos/juveniles with black capsule (BC) and without capsule (WC), exposed to two doses of UVB light (UVB-L and UVB-M), at 12, 24 and 49 days after UVB irradiation. No-UVB irradiated animals were used to determine the relative baseline (NUVB), represented by the dotted red line. Statistical significance was determined by Student's *t*-test (*n* = 3; *p < 0.05, **p < 0.01, ***p < 0.001, ^#^*p* < 0.0001, n.s. = no significative, n.d. = no data).

To evaluate the role of stress response genes in the defence against UVB stress in decapsulated (WC) and capsulated (BC) organisms, we studied the expression levels of Sof_Sod3, Sof_Hsp70 and Sof_Gst1 (figure 5).

Stress genes were more highly expressed than light-sensing genes (except Sof_r-Opsin1 in the eyes), with the highest level being about 6000 copies µl^−1^. On the other hand, the expression level of Sof_Hsp70 was similar in all four organs, while Sof_Gst1 was more expressed in the skin than in other organs, and the expression of Sof_Sod3 was only detected in the brain.

The basal expressions (NUVB) increased during the development in all cases except in the eye of capsulated embryos. The expression level of all stress genes was generally higher in decapsulated than in capsulated embryos. Expressions of all genes in all organs after UVB exposure (UVB-L and UVB-M) were higher than the basal expression, with one exception (Sof_Gst1 in optic lobes of juveniles under UVB-L treatment). In all cases, the expression level of these genes increased with the amount of UVB (NUVB < UVB-L < UVB-M) during the embryonic phase. It is noteworthy that the difference between UVB-L and UVB-M treatments was, in all cases, highly significant (from *p* < 0.001 to *p* < 0.0001), especially during days 12 and 24 of UVB exposure. By contrast, the capsulated embryos did not show such a significant response to the different treatments (from n.s. = *p* > 0.05 to *p* < 0.01).

#### 3.3.3. DNA repairs genes

**Figure 6.**
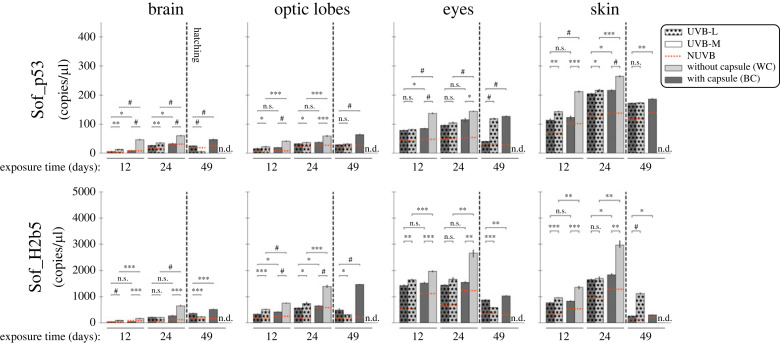
Gene expression levels (copies/µl, absolute quantification dPCR) of DNA repair-related genes (Sof_p53 and Sof_H2b5) in the four different tissues (brain, eyes, optic lobes and skin) from cuttlefish embryos/juveniles with black capsule (BC) and without capsule (WC), exposed to two doses of UVB light (UVB-L and UVB-M) at 12, 24 and 49 days of UVB exposure. No-UVB irradiated animals were used to determine relative baseline expression (NUVB), represented by the dotted red line. Statistical significance was determined by Student's *t*-test (*n* = 3; *p < 0.05, **p < 0.01, ***p < 0.001, ^#^*p* < 0.0001, n.s. = no significative, n.d. = no data).

DNA damage induced by UVB radiation was assessed using the expression levels of Sof_p53 and Sof_H2b5 genes in decapsulated (WC) and capsulated (BC) organisms (figure 6).

In the embryonic phase (12 and 24 days of UVB exposure), Sof_p53 and Sof_H2b5 were expressed in all organs with a higher level in the eye and skin than in the brain and optic lobes. However, Sof_H2b5 expression was 10-fold higher than the expression of Sof_p53, reaching 2966 ± 136.5 copies l^−1^ (skin, 24 days UVB-M decapsulated).

The basal expressions (NUVB) increased during the embryonic phase, although very slightly for Sof_p53 in the eyes. As for stress genes, levels of expression of Sof_p53 and Sof_H2b5 were higher in decapsulated embryos (exception: p53 in the eye). Expressions of the two genes were always higher when embryos were exposed to UVB. Moreover, a very significant difference appeared in all cases between UVB-L and UVB-M when embryos were decapsulated (from *p* < 0.001 to *p* < 0.0001), whereas the capsulated embryos did not show such a significant response to the variation of UVB radiation (from n.s. = *p*>0.05 to *p* < 0.05). Thus, the expression level of these genes increased with the amount of UVB (NUVB < UVB-L < UVB-M).

## 4. Discussion

The present study is the first to show the effects of continuous controlled UVB radiation exposure during the embryonic and juvenile phases on the cephalopod *Sepia officinalis*. It offers experimental data emphasizing the role of the black capsule as a protection against UVB radiation. In addition, we examined the relationship between the abnormalities observed and the induction of specific responses at molecular levels after UVB exposure. The daily UVB doses (8.0–24.8 kJ m^−2^) tested in this study were based on the data collected *in situ* and were representative of natural underwater daily UVB levels, where the high daily UVB doses detected underwater (0.1–0.3 m depth) were 40 kJ m^−2^ during July and 31 kJ m^−2^ during September 2021 in the mid-day light regimes. The daily UVB levels detected on the coast of Roscoff are similar to those reported underwater in other waters, such as the Atlantic Ocean, reaching daily UVB doses up to 44.5 kJ m^−2^ from December 2010 to 14 July 2011 [32] and Northwestern Mediterranean (41 kJ m^−2^) during June 2013 [33], demonstrating that the level of UVB radiation used in this study is of the same order of magnitude as the natural UVB found in the cuttlefish distribution area.

### 4.1. Effects of UVB on embryos

To our knowledge, this study is the first to propose a semi-quantitative assessment of the effects of UVB on the embryonic and juvenile stages in cephalopods.

Mortality and the development of abnormalities in cephalopod eggs and embryos, such as underdevelopment of the mantle and fins, as well as small body length, yolk fissure, and eye malformation found in this study, have been previously described. However, these abnormalities are usually associated with stress conditions such as extreme temperatures [30,31], episodes of hypoxia [31] or captivity [34]. Thus, UVB radiation can induce significant stress in cephalopods with results similar to other stressors, suggesting that UV-B light has many properties that make it a relevant stressor.

Previous work has shown that UV-B radiation can be associated with adverse effects on development and metamorphosis in several aquatic species (i.e. embryos of sea urchins [35], prawns [36] and fish [37]) and even cause considerable mortalities [38]. Moreover, the hypopigmentation phenomena (alteration of chromatophores) in embryos, as well as ocular structural and ultrastructural damage induced by UV-B radiation, have been observed in *Macrobrachium olfersi* shrimp [36] and *Clarias gariepinus* catfish [39]. The ocular damage and chromatophore alteration observed in this study may be due to an intense chromatic reaction after overexposure to UV-B radiation, as proposed by Nazari *et al*. [36], suggesting that pigment cell protection was unsuccessful.

Even at a high UVB irradiance (36 days, 24.8 kJ m^−2^ d^−2^; absolute UVB doses: 892.8 kJ m^−2^), successful embryonic development of *Sepia officinalis*, as evidenced by normal hatchlings, was observed in capsulated embryos. By contrast, abnormalities were observed in decapsulated embryos after five days of UVB-M treatment (14.4 kJ m^−2^ d^−1^; absolute UVB doses: 72 kJ m^−2^). Furthermore, these abnormalities increased in severity with the dose rate (compare UVB-M and UVB-H treatments) and the absolute dose of UV-B (as seen along the UVB-M treatment). These results suggest that the development of decapsulated embryos would be severely affected in the field. Therefore, the black *Sepia officinalis* egg capsule acts as a fundamental protective tool against the effects of UV-B.

Embryo encapsulation is described in many organisms as reducing embryonic vulnerability to various environmental stressors (including UV-B radiation), playing an essential role in embryo protection and development, thereby improving the prospects of juvenile success [7,38,40]. A recent study of the intertidal gastropod *A. monodon* egg capsule linked its ability to filter/attenuate different wavelengths to its morphology, thickness, and structure [38]. In cephalopods, the structure and physical properties of the egg capsule during embryonic development have not yet been fully documented [7,41]. In some sepiid species, including *Sepia officinalis*, melanin from the ink sac is incorporated into the capsule during egg deposition. Melanin and other photoprotective pigments with the same biological activity have been found in amphibian (e.g. *Rana temporaria* and *Xenopus* spp) and insect (e.g. *Podisus maculiventris*) eggs and have been associated with resistance to UV radiation [5,42]. Given the ecological characteristics of *Sepia officinalis* (eggs deposited in the intertidal area), the black egg capsule is critical for hatchling viability. We hypothesize that melanin and capsule structure play a role in the amount of UVB that can penetrate and reach the embryo. The ultrastructural changes that the egg capsule undergoes during embryonic development under natural and controlled stress conditions remain to be determined to obtain a complete picture of the capsule's ability to filter/absorb certain wavelengths.

### 4.2. Effects of UVB on juveniles

After hatching, juveniles were free to move around the aquarium and were directly exposed to UV-B radiation, especially the superficial tissues (skin, eyes). We have not studied their behaviour in detail, but we have noticed that they prefer to stay on the bottom and position themselves to limit their exposure. As a result, it is not easy to accurately assess their actual UVB exposure after hatching. Considering this restriction, the fact that no abnormalities were observed in either the control or UVB-L group throughout the experiment suggests that juveniles can withstand low doses of UVB radiation. The deleterious effect of UVB-M and UVB-H was pronounced, as demonstrated by the numerous abnormalities described in this work. These abnormalities increased in severity until the end of the experiments when more than 40% of the juveniles died. Among the abnormalities, a local increase in pigmentation has been observed in juveniles. Evidence of sunburn and skin hyperpigmentation following UV-B exposure has been observed in several aquatic organisms (i.e. the mollusc *Geomalacus maculosus* [43], the fish *Sparus aurata* [27] and amphibians such as *Physalaemus nattereri* [44] and *Taricha granulosa* [45]). Quantification of pigmentation levels in the juvenile skin of *S. officinalis* during UVB irradiation may bring elements to the understanding of the role of pigments in the photoprotection of cuttlefish.

To the best of our knowledge, this is the first study showing the effects of radiation on eyelid malformation in cephalopods and subsequent eye atrophy. Previous studies in fish have shown that chronic UVB exposure can cause the eyes’ nuclear opacities (cataracts) due to increased photooxidation activity [46,47]. Future work should further investigate the ultrastructural effects of UVB radiation on the eyes and skin in *S. officinalis*.

### 4.3. Gene expression in response to UVB exposure

UVB irradiation elicits a complex response involving multiple molecular pathways, as observed in aquatic animals, such as molluscs and fish [48–50]. Here, we focused on gene expression in four different tissues (brain, eyes, optic lobes and skin) in *S. officinalis* embryos exposed to NUVB, UVB-L and UVB-M for three different durations to assess the response and possible regulation in three categories of genes (light-sensitive, stress response and DNA repair) when the black capsule was present or absent.

In this study, we confirmed the results already observed by our group [10,51] regarding the expression of Sof_r-Opsin1. Under NUVB conditions, this gene is highly expressed during the terminal differentiation of the rhabdomeric photoreceptors of the retina (development stage 28–30, corresponding to 24 days of exposure in our experiment). The thinned capsule, therefore, appears to be permeable to UVB. However, the lower level of Sof_r-Opsin1expression under UVB-M compared to UVB-L suggests that the effect of UVB is limited by UVB damage, as already suggested by the morphological consequences we observed. Consequently, decapsulated embryos exposed to UVB always showed a reduced expression of Sof_Opsin1 compared with capsulated embryos.

Although the expression levels were very low, similar results were observed with Sof_Cry6. It is noteworthy that, compared to the NUVB condition, a higher expression of this gene was observed after 12 days of UVB exposure in the eye, confirming that this gene could play a role in the visual process and that cry6 is sensitive to UV in the eyes. Furthermore, the effect of UVB-L was enhanced in the absence of the capsule, underlining the filtering effect of the capsule. By contrast, Sof_Cry123 expression was not restricted to the eye and was continuously enhanced by UVB in a dose-dependent manner. This suggests that Sof_Cry123 is not restricted to the visual process, as Bonadè *et al*. [10] proposed, and may have a broader function than Sof_Cry6, such as a photolyase role in repairing damage caused by UVB irradiation, this role being suggested in other groups [12].

UVB-generated ROS can trigger an oxidative stress response, as previously demonstrated in several aquatic organisms [49,50,52]. In this study, we observed in all tissues that *S. officinalis* embryos without capsule exhibited a more pronounced oxidative stress response (Sof_Sod3, Sof_Hsp70 and Sof_Gst1), and DNA repair activity (Sof_p53 and Sof_H2b5) to UVB irradiation than those with capsules. These responses suggest that, in our experiments, *S. officinalis* embryos respond to high levels of ROS produced during UVB exposure, especially in the decapsulated group, thus demonstrating a protective role for the capsule.

We observed the most severe abnormalities and the highest expression levels of stress-related proteins in tissues directly exposed to UVB radiation (i.e. eyes and skin). Previous studies mainly focused on crustaceans [53–55] and fish [26,37,52] have shown that the increase of ROS concentration triggered by UVB radiation can induce morphological damage (e.g. desquamation, necrosis, sloughing of the skin, malformations) and consequently severe ocular diseases (e.g. cataract, glaucoma). Our experimental results in *S. officinalis* are consistent with these conclusions and underline the deleterious effects of UVB in this species. Furthermore, the reduced embryo length observed in decapsulated embryos under UVB-M treatment may be related to the expression of DNA repair genes. Overexpression of p53 delays cell division, which affects the total number of cells in a larva, and slows down the growth timing. Therefore, expression of the p53 pathway, if it does not lead to apoptosis causing larval mortality, may result in a smaller size at hatching, as observed in Atlantic cod larvae exposed to radiation and oxidative stress [16]. Furthermore, our results suggest that the presence of the capsule plays a critical role in attenuating the effects of UVB radiation on DNA and apoptosis.

Gene expressions show differences with and without capsule and morphological abnormalities developed only in organisms without capsule. This suggests that the capsule is an effective barrier against UVB. Nevertheless, UVB modulates gene expression in the embryo within the protective capsule: it is known that light passes through the capsule which stretches during development [51], and it is here assumed that the capsule does not block out all UVB rays, which may enhance/inhibit gene expression depending on the gene and the dose of UVB, regardless of the thickness of the capsule. As a result, molecular regulation, particularly of stress response and DNA repair genes, may occur and be able to prevent physiological dysfunction and subsequent morphological disorders.

The presence of physical protection (capsule) and an appropriate molecular response represent effective means of protection in *S. officinalis* and can be incorporated into photoprotective strategies. However, this example also highlights the need for studies at the level of gene expression to assess the effects of UVB, at stages before morphological changes are observed, even in the presence of proven effective protection.

## 5. Conclusion

The present study shows that the egg capsule effectively protects the embryonic development of the cuttlefish *Sepia officinalis* from the natural levels of UVB radiation that can typically be found in its natural distribution area. Furthermore, UVB experiments on embryos with and without a capsule indicate that the egg capsule is a permeable but biologically significant barrier to UVB radiation. Future research should focus on determining the capsule's spectral properties and structural changes during embryonic development to understand how this might affect the quality/quantity of light reaching the embryo. Finally, the protection provided by the capsule appears crucial for the survival of embryos exposed to UVB radiation, making *S. officinalis* an excellent model to study the effects of UVB changes in the context of global change.

## Data Availability

Data available from the Dryad Digital Repository: https://doi.org/10.5061/dryad.2280gb5xj [56]. Additional information is provided in electronic supplementary material [57].

## References

[1] Häder DP. 2011 Does enhanced solar UV-B radiation affect marine primary producers in their natural habitats? Photochem. Photobiol. **87**, 263-266. (10.1111/j.1751-1097.2011.00888.x) 21208211

[2] Vitt S, Bakker TCM, Rick IP. 2020 Differential investment in pre- and post-mating male sexual traits in response to prolonged exposure to ambient UVB radiation in a fish. Sci. Total Environ. **712**, 136341. (10.1016/j.scitotenv.2019.136341) 31931223

[3] Licht LE. 2003 Shedding light on ultraviolet radiation and amphibian embryos. Bioscience **53**, 551-561. (10.1641/0006-3568(2003)053[0551:SLOURA]2.0.CO;2)

[4] Epel D, Hemela K, Shickt M, Patton C. 1999 Development in the floating world: defenses of eggs and embryos against damage from UV radiation1. Am. Zool. **39**, 271-278. (10.1093/icb/39.2.271)

[5] Blaustein AR, Belden LK. 2003 Amphibian defenses against ultraviolet-B radiation. Evol. Dev. **5**, 89-97. (10.1046/j.1525-142X.2003.03014.x) 12492415

[6] Duellman WE, Trueb L. 1994 Biology of amphibians. Baltimore, MD: *Johns Hopkins University Press*.

[7] Cornet V, Henry J, Goux D, Duval E, Bernay B, Le Corguillé G, Corre E, Zatylny-Gaudin C, Schubert M. 2015 How egg case proteins can protect cuttlefish offspring? PLoS ONE **10**, 1-19. (10.1371/journal.pone.0132836) PMC4500399 26168161

[8] Guerra A. 2006 Ecology of Sepia officinalis. Vie Milieu **56**, 97-107.

[9] Boletzky SV, Andouche A, Aud-Ponticelli LB. 2016 A developmental table of embryogenesis in *Sepia officinalis*. Vie Milieu **66**, 11-23.

[10] Bonadè M, Ogura A, Corre E, Bassaglia Y, Bonnaud-Ponticelli L. 2020 Diversity of light sensing molecules and their expression during the embryogenesis of the cuttlefish (*Sepia officinalis*). Front. Physiol. **11**, 1-21. (10.3389/fphys.2020.521989) 33117186 PMC7553075

[11] Romagny S, Darmaillacq AS, Guibé M, Bellanger C, Dickel L. 2012 Feel, smell and see in an egg: emergence of perception and learning in an immature invertebrate, the cuttlefish embryo. J. Exp. Biol. **215**, 4125-4130. (10.1242/jeb.078295) 23136152

[12] Cronin TW, Bok MJ. 2016 Photoreception and vision in the ultraviolet. J. Exp. Biol. **219**, 2790-2801. (10.1242/jeb.128769) 27655820

[13] Kciuk M, Marciniak B, Mojzych M, Kontek R. 2020 Focus on UV-induced DNA damage and repair—disease relevance and protective strategies. Int. J. Mol. Sci. **21**, 1-33. (10.3390/ijms21197264) PMC7582305 33019598

[14] Yokawa K, Kagenishi T, Baluška F. 2016 UV-B induced generation of reactive oxygen species promotes formation of BFA-induced compartments in cells of Arabidopsis root apices. Front. Plant Sci. **6**, 1-10. (10.3389/fpls.2015.01162) PMC4710705 26793199

[15] Carrasco-Malio A, Díaz M, Mella M, Montoya MJ, Miranda A, Landaeta MF, Sánchez G, Hidalgo ME. 2014 Are the intertidal fish highly resistant to UV-B radiation? A study based on oxidative stress in *Girella laevifrons* (Kyphosidae). Ecotoxicol. Environ. Saf. **100**, 93-98. (10.1016/j.ecoenv.2013.07.030) 24238740

[16] Lesser MP, Farrell JH, Walker CW. 2001 Oxidative stress, DNA damage and p53 expression in the larvae of Atlantic cod (*Gadus morhua*) exposed to ultraviolet (290–400 nm) radiation. J. Exp. Biol **204**, 157-164. (10.1242/jeb.204.1.157) 11104719

[17] Halliwell B, Whiteman M. 2004 Measuring reactive species and oxidative damage *in vivo* and in cell culture: how should you do it and what do the results mean ? Br. J. Pharmacol. **142**, 231-255. (10.1038/sj.bjp.0705776) 15155533 PMC1574951

[18] Szyller J. 2021 Heat shock proteins in oxidative stress and ischemia / reperfusion injury and benefits from physical exercises : a review to the current knowledge. Oxid. Med. Cell. Long. **2021**, 1-2. (10.1155/2021/6678457) PMC7868165 33603951

[19] Dieterich A, Troschinski S, Schwarz S. 2015 Hsp70 and lipid peroxide levels following heat stress in *Xeropicta derbentina* (Krynicki 1836) (Gastropoda, Pulmonata) with regard to different colour morphs. Cell Stress and Chaperones **20**, 159-168. (10.1007/s12192-014-0534-3) 25108358 PMC4255243

[20] Coelho J, Court M, Otjacques E, Lopes VM, Paula JR, Repolho T, Diniz M, Rosa R. 2023 Effects of tidal emersion and marine heatwaves on cuttlefish early ontogeny. Mar. Biol. **170**, 1-12. (10.1007/s00227-022-04150-8)

[21] Herrera-Vásquez A et al. 2020 Transcription factor TGA2 is essential for UV-B stress tolerance controlling oxidative stress in Arabidopsis. *bioRxiv* 2020, 2020-2005.

[22] Zhou X, Tron VA, Li G, Trotter MJ. 1998 Heat shock transcription factor-1 regulates heat shock protein-72 expression in human keratinocytes exposed to ultraviolet B light. J. Invest. Dermatol. **111**, 194-198. (10.1046/j.1523-1747.1998.00266.x) 9699716

[23] Mao P, Wyrick JJ, Roberts SA, Smerdon MJ. 2017 Invited review UV-induced DNA damage and mutagenesis in chromatin. Photochem. Photobiol. **93**, 216-228. (10.1111/php.12646) 27716995 PMC5315636

[24] Montes-Rodríguez IM, Rodríguez-Pou Y, González-Méndez RR, Lopez-Garriga J, Ropelewski AJ, Cadilla CL. 2018 Characterization of histone genes from the bivalve lucina pectinata. Int. J. Environ. Res. Public Health **15**, 1-18. (10.3390/ijerph15102170) PMC6210712 30279399

[25] Vé R. 2012 E2F1 and p53 transcription factors as accessory factors for nucleotide excision repair. Int. J. Mol. Sci. **13**, 13 554-13 568. (10.3390/ijms131013554) PMC3497341 23202967

[26] Torres Nuñez E, Sobrino C, Neale PJ, Ceinos RM, Du S, Rotllant J. 2012 Molecular response to ultraviolet radiation exposure in fish embryos: implications for survival and morphological development. Photochem. Photobiol. **88**, 701-707. (10.1111/j.1751-1097.2012.01088.x) 22242699

[27] Alves RN, Mahamed AH, Alarcon JF, Al Suwailem A, Agustí S. 2020 Adverse effects of ultraviolet radiation on growth, behavior, skin condition, physiology, and immune function in gilthead seabream (*Sparus aurata*). Front. Mar. Sci. **7**, 1-20. (10.3389/fmars.2020.00306)32802822

[28] Rozen S, Skaletsky H. 2000 Primer3 on the WWW for general users and for biologist programmers. In Methods and protocols: methods in molecular biology (ed. JM Walker), pp. 365-386. Totawa, NJ: Humana Press. 10.1385/1-59259-192-2:365 10547847

[29] Seabold S, Perktold J. 2010 Statsmodels: econometric and statistical modeling with python. In Proc. 9th Python Sci. Conf., Austin, TX, 28 June - 3 July 2010, pp. 92-96. Austin, TX: SciPy.

[30] Gowland FC, Moltschaniwskyj NA, Steer MA. 2002 Description and quantification of developmental abnormalities in a natural *Sepioteuthis australis* spawning population (Mollusca: Cephalopoda). Mar. Ecol. Prog. Ser. **243**, 133-141. (10.3354/meps243133)

[31] Rosa R, Pimentel MS, Boavida-Portugal J, Teixeira T, Trübenbach K, Diniz M. 2012 Ocean warming enhances malformations, premature hatching, metabolic suppression and oxidative stress in the early life stages of a keystone squid. PLoS ONE **7**, e38282. (10.1371/journal.pone.0038282) 22701620 PMC3368925

[32] Garcia-Corral LS, Holding JM, Carrillo-de-Albornoz P, Steckbauer A, Pérez-Lorenzo M, Navarro N, Serret P, Duarte CM, Agusti S. 2017 Effects of UVB radiation on net community production in the upper global ocean. Glob. Ecol. Biogeogr. **26**, 54-64. (10.1111/geb.12513)

[33] Garcia-Corral LS, Martinez-Ayala J, Duarte CM, Agusti S. 2015 Experimental assessment of cumulative temperature and UV-B radiation effects on Mediterranean plankton metabolism. Front. Mar. Sci. **2**, 1-7. (10.3389/fmars.2015.00048)

[34] Jiménez-Prada P et al. 2014 Characterization of deformed hatchlings of *Octopus vulgaris* obtained under captivity from a small female. Fish. Res. **152**, 62-65. (10.1016/j.fishres.2013.08.017)

[35] Lamare MD, Barker MF, Lesser MP. 2007 *In situ* rates of DNA damage and abnormal development in Antarctic and non-Antarctic sea urchin embryos. Aquat. Biol **1**, 21-32. (10.3354/ab00003)

[36] Nazari EM, Ammar D, de Bem AF, Latini A, Müller YMR, Allodi S. 2010 Effects of environmental and artificial UV-B radiation on freshwater prawn *Macrobrachium olfersi* embryos. Aquat. Toxicol. **98**, 25-33. (10.1016/j.aquatox.2010.01.010) 20149463

[37] Alves RN, Agustí S. 2020 Effect of ultraviolet radiation (UVR) on the life stages of fish. Rev. Fish Biol. Fish. **30**, 335-372. (10.1007/s11160-020-09603-1)

[38] Cubillos VM, Salas-Yanquin LP, Büchner-Miranda JA, Ramírez F, Zabala MS, Averbuj A, Márquez F, Jaramillo HN, Chaparro OR. 2022 UV-R mitigation strategies in encapsulated embryos of the intertidal gastropod Acanthina monodon: a way to compensate for lack of parental care. Mar. Environ. Res. **180**, 105711. (10.1016/j.marenvres.2022.105711) 35933825

[39] Mahmoud UM, Mekkawy IAA, Sayed AEH. 2009 Ultraviolet radiation-A (366 nm) induced morphological and histological malformations during embryogenesis of *Clarias gariepinus* (Burchell, 1822). J. Photochem. Photobiol. B Biol **95**, 117-128. (10.1016/j.jphotobiol.2009.02.003) 19285878

[40] Rawlincs TA. 1999 Adaptations to physical stresses in the intertidal zone: the egg capsules of neogastropod molluscs. Am. Zool. **39**, 230-243. (10.1093/icb/39.2.230)

[41] Boletzky S. 1986 Encapsulation of cephalopod embryos - a search for functional correlations. Am. Malacol. Bull. **4**, 217-227.

[42] Abram PK, Guerra-Grenier E, Després-Einspenner ML, Ito S, Wakamatsu K, Boivin G, Brodeur J. 2015 An insect with selective control of egg coloration. Curr. Biol. **25**, 2007-2011. (10.1016/j.cub.2015.06.010) 26212882

[43] O'Hanlon A, Feeney K, Dockery P, Gormally MJ. 2017 Quantifying phenotype-environment matching in the protected Kerry spotted slug (Mollusca: Gastropoda) using digital photography: exposure to UV radiation determines cryptic colour morphs. Front. Zool. **14**, 1-12. (10.1186/s12983-017-0218-9) 28702067 PMC5504635

[44] Franco-belussi L, Sko HN. 2016 Internal pigment cells respond to external UV radiation in frogs. J. Exp. Biol. **19**, 1378-1383. (10.1242/jeb.134973) 26944494

[45] Belden LK, Blaustein AR. 2002 UV-B induced skin darkening in larval salamanders does not prevent sublethal effects of exposure on growth, vol. 2002, pp. 748-754. American Society of Ichthyologists and Herpetologists (ASIH) (https://www.jstor.org/stable/1448155).

[46] Cullen AP. 1993 Damage to the rainbow trout (*Oncorhyncus mykiss*) lens following an acute dose of UVB. Eye Eye **12**, 97-106. (10.3109/02713689308999477) 8449030

[47] Cullen AP, Monteith-Mcmaster CA, Sivak JG. 1994 Lenticular changes in rainbow trout following chronic exposure to UV radiation. Curr. Eye Res. **13**, 731-737. (10.3109/02713689409047007) 7842722

[48] Russo R, Bonaventura R, Matranga V. 2014 Time- and dose-dependent gene expression in sea urchin embryos exposed to UVB. Mar. Environ. Res. **93**, 85-92. (10.1016/j.marenvres.2013.08.006) 24011617

[49] Bonaventura R, Poma V, Russo R, Zito F, Matranga V. 2006 Effects of UV-B radiation on development and hsp70 expression in sea urchin cleavage embryos. Mar. Biol. **149**, 79-86. (10.1007/s00227-005-0213-0)

[50] Alves RN, Agustí S. 2021 Oxidative stress in tissues of gilthead seabream (*Sparus aurata*) and European seabass (*Dicentrarchus labrax*) juveniles exposed to ultraviolet-B radiation. J. Photochem. Photobiol. **8**, 100070. (10.1016/j.jpap.2021.100070)

[51] Imarazene B, Andouche A, Bassaglia Y, Lopez PJ, Bonnaud-Ponticelli L. 2017 Eye development in Sepia officinalis embryo: What the uncommon gene expression profiles tell us about eye evolution. Front. Physiol. **8**, 613. (10.3389/fphys.2017.00613) 28883798 PMC5573735

[52] Icoglu Aksakal F, Ciltas A. 2018 The impact of ultraviolet B (UV-B) radiation in combination with different temperatures in the early life stage of zebrafish (*Danio rerio*). Photochem. Photobiol. Sci. **17**, 35-41. (10.1039/c7pp00236j) 29147715

[53] De Moraes Vaz Batista Filgueira D, Guterres LP, De Souza Votto AP, Vargas MA, Boyle RT, Trindade GS, Nery LEM. 2010 Nitric oxide-dependent pigment migration induced by ultraviolet radiation in retinal pigment cells of the crab *Neohelice granulata*. Photochem. Photobiol **86**, 1278-1284. (10.1111/j.1751-1097.2010.00787.x) 21091482

[54] Kim BM, Rhee JS, Lee KW, Kim MJ, Shin KH, Lee SJ, Lee YM, Lee JS. 2015 UV-B radiation-induced oxidative stress and p38 signaling pathway involvement in the benthic copepod *Tigriopus japonicus*. Comp. Biochem. Physiol. Part - C Toxicol. Pharmacol. **167**, 15-23. (10.1016/j.cbpc.2014.08.003) 25152408

[55] Vaz VVA, Jardim da Silva L, Geihs MA, Maciel FE, Nery LEM, Vargas MA. 2020 Single and repeated low-dose UVB radiation exposures affect the visual system. J. Photochem. Photobiol. B Biol. **209**, 111941. (10.1016/j.jphotobiol.2020.111941) 32629396

[56] Molina-Carrillo L, Bassaglia Y, Schires G, Bonnaud-Ponticelli L. 2023 Data from: Does the egg capsule protect against chronic UV-B radiation? A study based on encapsulated and decapsulated embryos of cuttlefish *Sepia officinalis*. Dryad Digital Repository. (10.5061/dryad.2280gb5xj) PMC10354468 37476507

[57] Molina-Carrillo L, Bassaglia Y, Schires G, Bonnaud-Ponticelli L. 2023 Does the egg capsule protect against chronic UV-B radiation? A study based on encapsulated and decapsulated embryos of cuttlefish *Sepia officinalis*. Figshare. (10.6084/m9.figshare.c.6742186) PMC10354468 37476507

